# Dynamic Modeling and Analysis of Rotary Joints with Coupled Bearing Tilt-Misalignment Faults

**DOI:** 10.3390/e27111123

**Published:** 2025-10-31

**Authors:** Jun Lu, Zixiang Zhu, Jie Ji, Yichao Yang, Xueyang Miao, Xiaoan Yan, Qinghua Liu

**Affiliations:** 1 Jiangsu NARI Power Electric Co., Ltd., Nanjing 211106, China; 13851582074@163.com; 2 College of Mechanical and Electronic Engineering, Nanjing Forestry University, Nanjing 210037, China

**Keywords:** rotary joint, bearing tilt-misalignment coupling fault, nonlinear dynamics, hertzian contact theory, composite fault

## Abstract

This study systematically analyzes the dynamic behavior of bearing tilt-misalignment coupling faults in rotary joints and establishes a high-fidelity nonlinear dynamic model for a dual-support bearing–rotor system. By integrating Hertzian contact theory, the nonlinear contact forces induced by the tilt of the inner/outer rings and axial misalignment are considered, and expressions for bearing forces incorporating time-varying stiffness and radial clearance are derived. The system’s vibration response is solved using the Newmark-β numerical integration method. This study reveals the influence of tilt angle and misalignment magnitude on contact forces, vibration patterns, and fault characteristic frequencies, demonstrating that the system exhibits multi-frequency harmonic characteristics under misalignment conditions, with vibration amplitudes increasing nonlinearly with the degree of misalignment. Furthermore, dynamic models for single-point faults (inner/outer ring) and composite faults are constructed, and Gaussian filtering technology is employed to simulate defect surface roughness, analyzing the modulation effects of faults on spectral characteristics. Experimental validation confirms that the theoretical model effectively captures actual vibration features, providing a theoretical foundation for health monitoring and intelligent diagnosis of rotary joints.

## 1. Introduction

As a critical component for continuous fluid or gas transfer between static and rotating systems, the health condition of rolling bearings in rotary joints directly affects operational stability and service life. Due to inevitable assembly errors, structural deformations, and uneven loads in practical applications, rotary joint bearings are highly susceptible to tilt-misalignment coupling during long-term operation. This misalignment not only disrupts the nominal force distribution but also significantly alters the bearing’s internal contact characteristics and dynamic behavior, leading to increased vibration amplitudes, localized overloading, and fatigue damage. Analyzing the dynamic characteristics of rotary joint bearings under tilt-misalignment coupling is crucial for understanding fault mechanisms, enabling early diagnosis, and improving health monitoring [1].

Existing research on rolling bearing misalignment primarily focuses on single-fault modes or simplified models, which may not fully capture the nonlinear coupled dynamics in real rotary joint systems. Particularly when tilt misalignment coexists with axial offset, the system’s force boundary conditions and vibration responses exhibit stronger nonlinearity and multi-frequency characteristics, making conventional modeling approaches inadequate for accurately tracking dynamic evolution. Therefore, it is essential to develop a high-fidelity model that accounts for inner/outer ring tilt, axial misalignment, and multi-source nonlinearities to characterize the influence of coupled tilt and misalignment on dynamic behavior [2,3].

In studies related to bearing misalignment, researchers have conducted a series of investigations on the effects of misalignment on the static characteristics of bearings, primarily focusing on contact characteristics [4,5,6,7,8], bearing stiffness [9,10], and fatigue life [9,10,11]. In the research on the dynamic characteristics of rotor systems with misaligned rolling bearings, Yi et al. [12] proposed a nonlinear force model considering parallel misalignment in rolling bearings. This model was applied to the finite element model (FEM) of a machine tool spindle to investigate the influence of bearing misalignment on the spindle’s dynamic behavior and amplitude. Wang et al. [13], proposed a five-degree-of-freedom nonlinear dynamic model for a ball bearing–rotor system that considers both bearing misalignment and coupling misalignment, addressing the gap in existing studies that typically neglect bearing excitation effects. Their model incorporates Timoshenko beam theory, coupling misalignment forces, and rotor imbalance to analyze system vibrations, demonstrating that bearing misalignment significantly influences low-speed dynamics while coupling effects dominate at higher speeds. The study also reveals that misalignment alters bearing contact angles, clearance, and cage interactions, while increasing critical speeds and axial vibrations, providing insights for rotor system fault diagnosis. Parmar et al. [14] derived a nonlinear force expression for a double-row spherical rolling bearing considering angular misalignment and outer race defects. Wen et al. [15] proposed a stiffness model for ball bearings that considers both parallel and angular misalignments and analyzed the dynamic behavior of a rigid rotor under these two misalignment conditions.

Based on the above studies, as the complexity of the research objects increases, dynamic modeling becomes increasingly challenging, yet several problems remain unresolved. A single fault in one component of rotating machinery can induce faults in other parts and types, eventually leading to multiple compound faults [16,17,18,19,20,21]. Current research on the dynamics of rotating machinery, both domestically and internationally, primarily focuses on single faults. Understanding the coupling between fault characteristics and dynamic response during compound fault evolution is critical for improving bearing lifespan and enabling intelligent fault diagnosis [22,23,24,25,26,27]. Based on the aforementioned research, this study establishes a finite element dynamic model of a double-support bearing–rotor coupled system. By analyzing the system’s mechanical characteristics, the corresponding nonlinear motion differential equations are derived.

This study begins with tilt misalignment caused by interference fits and assembly errors, establishing a finite element dynamic model for a dual-bearing-single-rotor system. By incorporating a nonlinear Hertzian contact model, the force-contact angle relationship for each rolling element under tilt-misalignment coupling is derived, and bearing force expressions considering time-varying stiffness and radial clearance are formulated. Additionally, the additional loads induced by shaft inclination are integrated into the governing differential equations. Using the Newmark-β numerical integration method, the system’s vibration response is solved, and the effects of tilt angle and misalignment magnitude on contact forces, vibration patterns, and fault characteristic frequencies are analyzed. This work provides a theoretical foundation for future research on fault feature extraction and intelligent diagnosis of rotary joint bearings.

The model primarily focuses on the nonlinear dynamics induced by bearing tilt-misalignment and composite faults, with the following key assumptions: (1) The bearing internal contact follows Hertzian theory under ideal material and geometric conditions; (2) Tilt misalignment is modeled as static angular deviations that systematically alter the contact angle and effective radial clearance as functions of rolling element position; (3) Localized raceway defects are characterized by predefined geometric profiles (arc segments) superimposed with Gaussian-filtered random roughness; (4) Shaft misalignment loads are derived from geometric relationships and integrated as static excitations. The model is specifically developed for double-support bearing–rotor systems in rotary joints, applicable for analyzing vibration characteristics and fault mechanisms in low-to-moderate speed regimes where these nonlinearities dominate, while explicitly excluding complex lubrication dynamics and thermal effects.

## 2. Dynamic Analysis of the System Model

### 2.1. Generation Mechanism of Bearing Inner/Outer Ring Tilt and Misalignment

**Figure 1:**
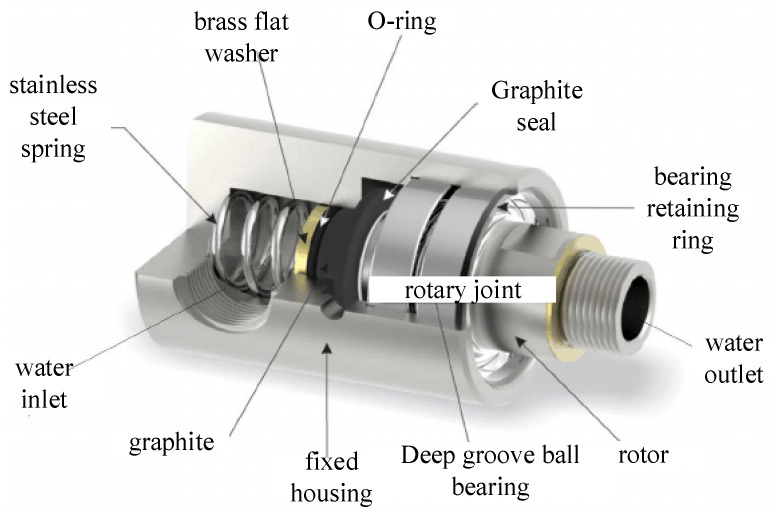
Structure of a rotary joint.

During the operation of bearings, due to factors such as interference fit and assembly accuracy, the inner and outer rings often become misaligned and tilted. This situation leads to relative motion between the inner and outer rings of the bearing, generating additional loads and nonlinear forces, which in turn affect the overall dynamic characteristics of the bearing [28]. This study first considers the misalignment and tilt of the inner and outer rings of the bearing caused by interference fit and establishes the relevant mathematical model. Figure 1 shows the structural diagram of the rotary joint, mainly composed of the housing, rotor, seal, bearing, etc.

**Figure 2:**
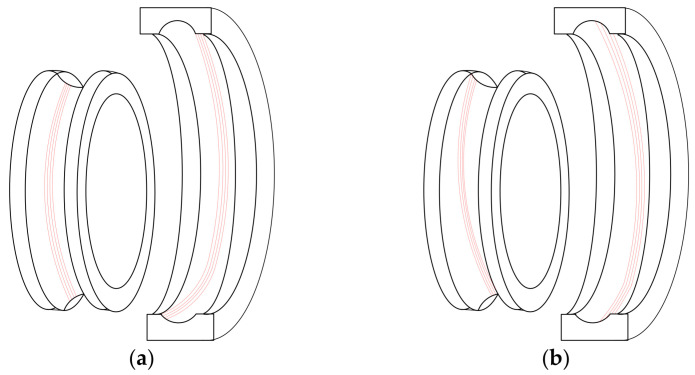
Eccentricity of bad contact marks: (**a**) Outer ring eccentricity; (**b**) Inner ring eccentricity.

During the operation of the bearing, the contact between the balls and the raceway will form obvious contact marks on the raceway surface. When the inner and outer rings of the bearing are misaligned due to poor centering and produce tilt or eccentricity, this misalignment condition will cause changes in the distribution of contact stress. If the bearing operates under such conditions for a long time, the contact marks on the raceway surface will show a significant offset phenomenon, as shown in Figure 2. To deeply study the impact of poor centering of the bearing on its operational performance, it is necessary to establish a corresponding dynamic model, analyze its dynamic response characteristics, and thus enable early human intervention when such a situation occurs.

### 2.2. Rolling Body Contact Model and Nonlinear Mechanical Properties

**Figure 3:**
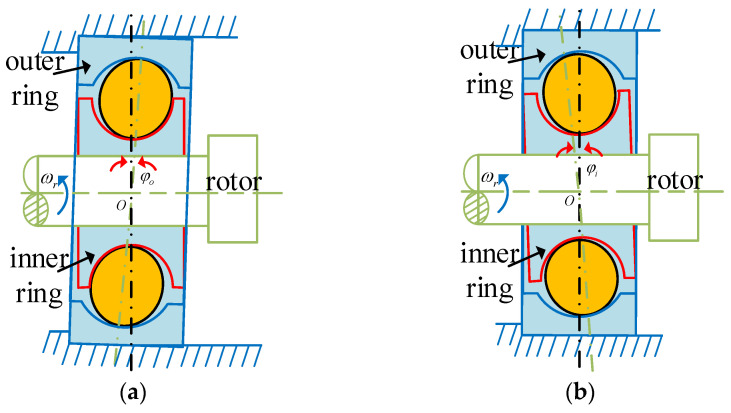
Internal dimensions of the bearing: (**a**) Outer ring misalignment; (**b**) Inner ring misalignment.

In the rotary joint, the unbalanced phenomenon of the bearings mainly results from the combined effects of shaft system deformation, assembly errors, and interference fit. Depending on the different positions of the deviation, it can be classified into two basic forms: inner ring deviation type and outer ring deviation type. These two unbalanced patterns will affect the dynamic characteristics of the system by altering the distribution of the bearing contact load. As shown in Figure 3.

**Figure 4:**
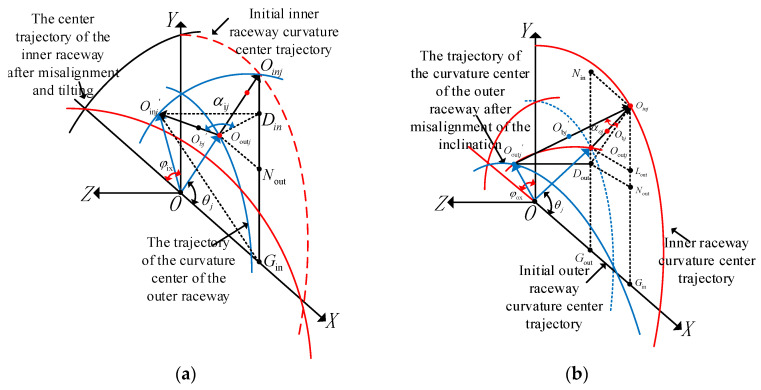
Tilt geometry between the inner and outer rings of the bearing: (**a**) The inner ring is not properly aligned; (**b**) The outer ring is not aligned properly.

To model assembly-induced misalignment, we assume the inner ring deflects along the x axis with an inclination angle. The inclination angle is denoted as φ_ix_, Let the angular position of the j-th rolling body be θ_j_, The initial center position is O_bj_, The corresponding inner raceway curvature center trajectory point is O_inj_, As shown in Figure 2, Figure 3 and Figure 4, the geometric relationship is as follows. When the inner ring deflects, the trajectory of the curvature center of the inner raceway undergoes displacement, causing the contact point position of the j-th rolling element to be updated to O_bj_’. The corresponding inner raceway trajectory points have changed to O_inj_’. Based on the geometric relationship shown in Figure 4, the expressions for the lengths of the radii of the curvature centers of the inner and outer raceways can be derived as follows:

(1) $$\left|\vec{OO_{inj}}\right|=r_{b}+r_{in},\left|\vec{OO_{outj}}\right|=R_{b}-r_{out}$$

In the formula, the curvature radii of the inner and outer raceways are, respectively, r_in_ and r_out_. By introducing the dimensionless inner/outer raceway curvature radius coefficients fi/o, the raceway curvature radius can be expressed as a function of the ball diameter d_b_: r_in_ = f_i_d_b_; rout = f_o_d_b_.

To enhance the universality of the model, it is necessary to further consider the deflection effect of the inner ring in the y axis direction. Assuming that the tilt in this direction does not cause the centering, deflection angle is φ_iy_. Then the system will exhibit the following geometric characteristics [29]:

(2) $$\left|\vec{D_{in}O_{{inj}^{\prime }}}\right|=\left(r_{b}+r_{in}\right)\left[\sin\theta_{j}\sin\varphi_{ix}-\cos\theta_{j}\sin\varphi_{iy}\right]$$

(3) $$\left|\vec{D_{in}O_{inj}}\right|=(r_{b}+r_{\mathrm{in}})\sqrt{\sin^{2}\theta_{j}{\left(1-\cos\varphi_{\mathrm{ix}}\right)}^{2}+\cos^{2}\theta_{j}{\left(1-\cos\varphi_{\mathrm{iy}}\right)}^{2}}$$

(4) $$\left|\vec{O_{\mathrm{out}j}N_{\mathrm{out}}}\right|=\left|\vec{O_{\mathrm{out}j}O_{\mathrm{in}j}}\right|\cos\theta_{j}=\;(r_{\mathrm{in}}+r_{\mathrm{out}}-d_{b}-c_{0})\cos\theta_{j}$$

(5) $$\begin{array}{l} \left|\vec{O_{\mathrm{out}j}D_{\mathrm{in}}}\right|=\sqrt{{\left(\left|\vec{O_{\mathrm{out}j}N_{\mathrm{out}}}\right|\right)}^{2}+{\left(\left|\vec{N_{\mathrm{out}}D_{\mathrm{in}}}\right|\right)}^{2}}=\\ \left\{{\left[\left(r_{\mathrm{in}}+r_{\mathrm{out}}-d_{b}-c_{0}\right)\cos\theta_{j}\right]}^{2}+\right.{\left[\begin{array}{c} (r_{\mathrm{in}}+r_{\mathrm{out}}-d_{b}-c_{0})\sin\theta_{j}-(r_{b}+r_{\mathrm{in}})B_{i} \end{array}\right]}^{\frac{1}{2}} \end{array}$$

Based on the geometric configuration shown in the diagram, the contact α_ij_ formed by the j-th rolling element at the angular position θ_j_ due to the skew of the bearing axis can be derived. Its mathematical expression is [29]:

(6) $$\alpha_{ij}=\arctan\left(\frac{\left|\vec{D_{\mathrm{in}}{O_{\mathrm{in}j}}^{\prime }}\right|}{\left|\vec{O_{\mathrm{out}j}D_{\mathrm{in}}}\right|}\right)$$

The normal clearance at the j-th ball after misalignment is defined as Δ_0ij_. Since the curvature radius of the bearing raceway is much larger than the normal clearance, referring to Figure 4a, the radial clearance of the bearing at the angular position of the j-th ball c’_0ij_ can be expressed as:

(7) $$c_{0ij}^{\prime }=\frac{\Delta_{ij}}{\cos\alpha_{ij}}=\frac{r_{\mathrm{in}}+r_{\mathrm{out}}-d_{b}-\sqrt{{\left(\left|\vec{D_{\mathrm{in}}{O_{\mathrm{in}j}}^{\prime }}\right|\right)}^{2}+{\left(\left|\vec{O_{\mathrm{out}j}D_{\mathrm{in}}}\right|\right)}^{2}}}{\cos\left[\arctan\left(\frac{\left|\vec{D_{\mathrm{in}}{O_{\mathrm{in}j}}^{\prime }}\right|}{\left|\vec{O_{\mathrm{out}j}D_{\mathrm{in}}}\right|}\right)\right]}$$

Finally, substitute Equations (2) and (5) into Equations (6) and (7), and a_0_= (f_i_ + f_o_ − 1)d_bo_. The generalized expression for the contact angle and the bearing clearance after the bearing inner ring undergoes any-directional tilt and misalignment can be obtained as follows:

(8) $$\alpha_{ij}=\arctan\left(\frac{\left(r_{b}+f_{i}\times d_{b}\right)A_{i}}{\sqrt{{\left(a_{0}-c_{0}\right)}^{2}\cos^{2}\theta_{j}+{\left[\left(a_{0}-c_{0}\right)\sin\theta_{j}-\left(r_{b}+f_{i}\times d_{b}\right)B_{i}\right]}^{2}}}\right)$$

(9) $$c_{0ij}^{\prime }=\frac{a_{0}-\sqrt{{\left(r_{b}+f_{i}\times d_{b}\right)}^{2}A_{i}^{2}+{\left(a_{0}-c_{0}\right)}^{2}\cos^{2}\theta_{j}+{\left[\left(a_{0}-c_{0}\right)\sin\theta_{j}-\left(r_{b}+f_{i}\times d_{b}\right)B_{i}\right]}^{2}}}{\cos\left[\arctan\left(\frac{\left(r_{b}+f_{i}\times d_{b}\right)A_{i}}{\sqrt{{\left(a_{0}-c_{0}\right)}^{2}\cos^{2}\theta_{j}+{\left[\left(a_{0}-c_{0}\right)\sin\theta_{j}-\left(r_{b}+f_{i}\times d_{b}\right)B_{i}\right]}^{2}}}\right)\right]}$$

In Equations (5), (8) and (9):

(10) $$A_{i}=\sin\theta_{j}\sin\varphi_{\mathrm{ix}}-\cos\theta_{j}\sin\varphi_{\mathrm{iy}}$$

(11) $$B_{i}=\sqrt{\sin^{2}\theta_{j}{\left(1-\cos\varphi_{\mathrm{ix}}\right)}^{2}+\cos^{2}\theta_{j}{\left(1-\cos\varphi_{\mathrm{iy}}\right)}^{2}}$$

Similarly, when considering the assembly problem that leads to the skewed condition of the outer ring, let the deflection angles of the outer ring in the x and y directions be, respectively, φ_ox_ and φ_oy_, its geometric configuration is shown in Figure 4b. The outer ring deflection will cause the deviation of the curvature center trajectory of the outer raceway, and the contact point of the outer raceway corresponding to the j-th rolling element will be updated to O’_outj_. Based on the geometric constraint relationship shown in Figure 4b, using the same derivation method as before, the analytical expressions for the generalized contact angle α_oj_ and the equivalent bearing clearance c’_0oj_ under the condition of any-direction misalignment of the outer ring can be obtained:

(12) $$a_{oj}=\arctan\left(\frac{\left(R_{b}-f_{o}\times d_{o}\right)A_{o}}{\sqrt{{\left(a_{o}-c_{o}\right)}^{2}\cos^{2}\theta_{j}+{\left[(a_{o}-c_{o})\sin\theta_{j}+(R_{b}-f_{o}\times d_{o})B_{o}\right]}^{2}}}\right)$$

(13) $$c_{0oj}^{\prime }=\frac{a_{0}-\sqrt{{\left(R_{b}-f_{o}\times d_{b}\right)}^{2}A_{o}^{2}+{\left(a_{0}-c_{0}\right)}^{2}\cos^{2}\theta_{j}+{\left[\left(a_{0}-c_{0}\right)\sin\theta_{j}-\left(R_{b}-f_{o}\times d_{b}\right)B_{o}\right]}^{2}}}{\cos\left[\arctan\left(\frac{(R_{b}-f_{o}\times d_{b})A_{o}}{\sqrt{{\left(a_{0}-c_{0}\right)}^{2}\cos^{2}\theta_{j}+{\left[\left(a_{0}-c_{0}\right)\sin\theta_{j}-\left(R_{b}-f_{o}\times d_{b}\right)B_{o}\right]}^{2}}}\right)\right]}$$

In Equations (12) and (13):

(14) $$A_{o}=\sin\theta_{j}\sin\varphi_{ox}-\cos\theta_{j}\sin\varphi_{oy}$$

(15) $$B_{o}=\sqrt{\sin^{2}\theta_{j}{\left(1-\cos\varphi_{\mathrm{ox}}\right)}^{2}+\cos^{2}\theta_{j}{\left(1-\cos\varphi_{\mathrm{oy}}\right)}^{2}}$$

The derived formula indicates that when the skew misalignment of the inner/outer ring in a deep groove ball bearing, the actual working clearance at the position of the j-th rolling element is a function of multiple parameters. The influencing factors include: the misalignment displacement α_i/oj_, (r_i/o_), (f_i/o_), d_b_ and c_0_. If the initial phase angle of the first rolling element is defined as zero, according to the bearing assembly coordinate system established in this paper, the angular position θ_j_ of the j-th rolling element can be determined by the following formula:

(16) $$\theta_{j}=\frac{\omega r_{b}t}{R_{b}+r_{b}}+\frac{2\pi }{N_{b}}(j-1)$$

Based on the analytical relationships of Equations (8)–(16), the elastic deformation amount of the contact surface between the j-th rolling element and the raceway of the bearing under the unbalanced condition can be determined:

(17) $$\delta_{j}=x\cos\theta_{j}+y\sin\theta_{j}-c_{0n}^{\prime }$$

In the formula, x and y represent the vibration displacement components of the inner ring center in the horizontal and vertical directions, respectively; the subscript n is used to distinguish between the two working conditions: inner ring misalignment (ij) and outer ring misalignment (o_j_).

Based on the nonlinear Hertz contact theory, the interaction between the rolling elements and the raceways can only generate normal contact stresses; that is, there is an effective contact force only when δ_j_ > 0. By introducing the Heaviside step function H(δ_j_), the nonlinear bearing contact forces caused by the misalignment can be expressed in the x and y directions as follows:

(18) $$\left\{\begin{array}{c} F_{bx}=-\sum_{j=1}^{N_{b}}c_{bj}\delta_{j}^{1.5}H(\delta_{j})\cos\alpha_{n}\cos\theta_{j}\\ F_{by}=-\sum_{j=1}^{N_{b}}c_{bj}\delta_{j}^{1.5}H(\delta_{j})\cos\alpha_{n}\sin\theta_{j} \end{array}\right.$$

Among them, the parameter c_bj_ represents the Hertz contact stiffness coefficient of the j-th rolling body. Its numerical characteristics depend on the material properties (such as elastic modulus, Poisson’s ratio) and geometric configuration (curvature radius, contact angle, etc.) of the contact pair.

### 2.3. The Reactive Force Caused by the Misalignment of the Axis

**Figure 5:**
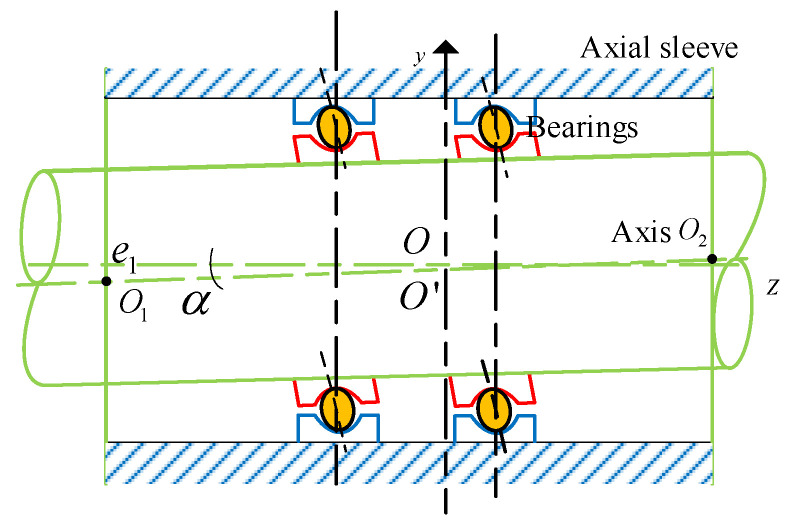
Models with inclination angle and inclination error of inner and outer rings.

Figure 5 shows the model of a rolling bearing that simultaneously has an axial inclination angle α and an error in the inclination of the inner and outer rings of the bearing. O represents the center of the shaft, O′ is the center of the shaft neck at the midpoint of the length direction of the bearing, and the eccentric distance e = |OO_1_|. The radius of the shaft sleeve is R, and the radius of the shaft is r. Let the inclination angle between the shaft and the shaft sleeve be α, and O_1_, O_2_ are the centers of the shaft at the sections where it is located on the end faces of the shaft sleeve.

When the journal of the shaft is tilted within the bearing hole, as long as the eccentricity e at each cross-section along the length direction of the bearing is calculated based on the tilt angle, since the bearing is divided into n sections along the length direction, and given the known inclination angle α, it is further easy to obtain the eccentricity e_j_ of each node at the cross-section as follows:

(19) $$e_{j}=\frac{L}{2}\tan\alpha -(j-1)\frac{L}{n}\tan\alpha =\frac{L}{n}\cdot \tan\alpha \cdot (\frac{n}{2}-j+1)$$

**Figure 6:**
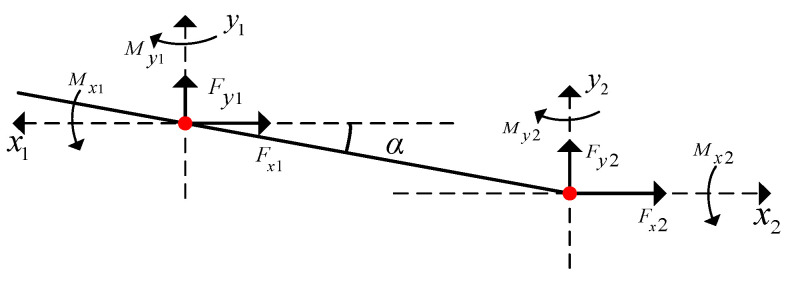
Axis tilt misalignment coupling coordinate system.

As shown in Figure 6, an axis misalignment coordinate system is established. For the rotary joint, there is an axis tilt and misalignment between the two bearings.

(20) $$\left\{\begin{array}{c} F_{x2}=\frac{T_{q}\sin\alpha }{e_{j}}\\ F_{y2}=\frac{T_{q}\cos\alpha }{e_{j}} \end{array}\right.$$

It can be seen that the reaction forces Fx2 and Fy2 are functions of the angular displacement and the lateral translation displacement. Assume that Z1 is the driving side, as shown in the figure, T_q_ is applied, and the rotation direction is consistent with the applied torque. For the shaft, the static load can be decomposed into a series of dynamic periodic forces that cause vibration at the coupled nodes of the finite element model. The dynamic excitation force and dynamic bending moment are regarded as the excitation [30]. Assuming that all the reaction forces caused by the misalignment of the axis act at the node j of the system, the force expression is:

(21) $$F_{c}=\left\{\begin{array}{c} F_{x2}(\sin(\omega t)+\sin(2\omega t)+\sin(3\omega t)+\sin(4\omega t))\\ F_{y2}(\cos(\omega t)+\cos(2\omega t)+\cos(3\omega t)+\cos(4\omega t)) \end{array}\right.$$

## 3. Dynamics Modeling and Simulation

### 3.1. System Finite Element Model

This study focuses on the analysis of the double-support bearing–rotor system. The specific operating conditions and structural parameters of this system are detailed in Table 1 and Table 2. The dynamic response of the system is solved using the Newmark-β time-domain integration algorithm, and a systematic analysis of the dynamic characteristics of the numerical simulation results is conducted [31]. Specifically, the parameters were selected as γ = 0.5 and β = 0.25 to ensure unconditional stability of our nonlinear system. Meanwhile, the time step was determined through convergence analysis to guarantee numerical accuracy while maintaining computational efficiency. The differential equation of motion for the double-bearing-single-rotor system is:

(22) $$M\ddot{q}+(C+\omega G)\dot{q}+Kq=F_{e}+F_{b}+F_{c}$$

In the formula, M represents the element mass matrix; C is the element damping matrix; G is the element gyroscopic matrix; K is the element stiffness matrix; F_b_ is the nonlinear bearing contact force of the bearing; F_e_ is the unbalanced load; F_c_ is all the reaction forces caused by the misalignment of the axis.

### 3.2. Changes in the Dynamic Characteristics of the System

**Figure 7:**
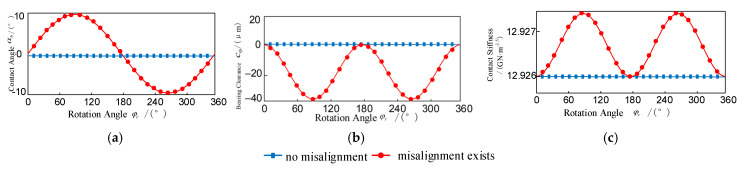
The contact angle, bearing clearance, and contact stiffness vary with the angle of rotation: (**a**) Contact angle; (**b**) Radial clearance; (**c**) Contact stiffness.

As shown in Figure 7, the inner/outer ring skewing causes the key mechanical parameters of the bearing to exhibit dynamic variation characteristics: the working clearance, contact angle, and contact stiffness all deviate from the constant values, showing obvious periodic fluctuations. This skewing condition will form two symmetrical negative clearance areas in the circumferential direction of the bearing, corresponding to the unique bearing characteristics of dual pressure zones. During the circular motion of the rolling elements, each rolling element will undergo periodic mechanical state transitions of “loading-unloading3”. Although the average contact angle of the rolling elements for one rotation remains zero, the instantaneous contact angle can change by ±10°. This contact angle oscillation phenomenon causes the contact stiffness of each rolling element to present a bi-frequency periodic fluctuation characteristic, that is, there are two extreme stiffness value changes in each rotation cycle. According to the aforementioned theoretical model analysis, this dynamic characteristic is caused by the geometric parameter coupling effect resulting from the misalignment of the bearing.

Under operating conditions where the bearing inner ring rotates at a constant angular velocity ω, the revolution speed ω_j_ of each rolling element is fundamentally determined by the instantaneous contact angle αj. The spatial non-uniform distribution of contact angles induced by assembly misalignment leads to differentiated revolution speeds among rolling elements. This kinematic incompatibility causes rotational speed mismatch between certain rolling elements and the cage, thereby generating periodic impact loads. Both theoretical analysis and experimental observations demonstrate that such high-frequency elastic collision effects constitute one of the primary mechanisms responsible for cumulative fatigue damage in cage structures.

**Figure 8:**
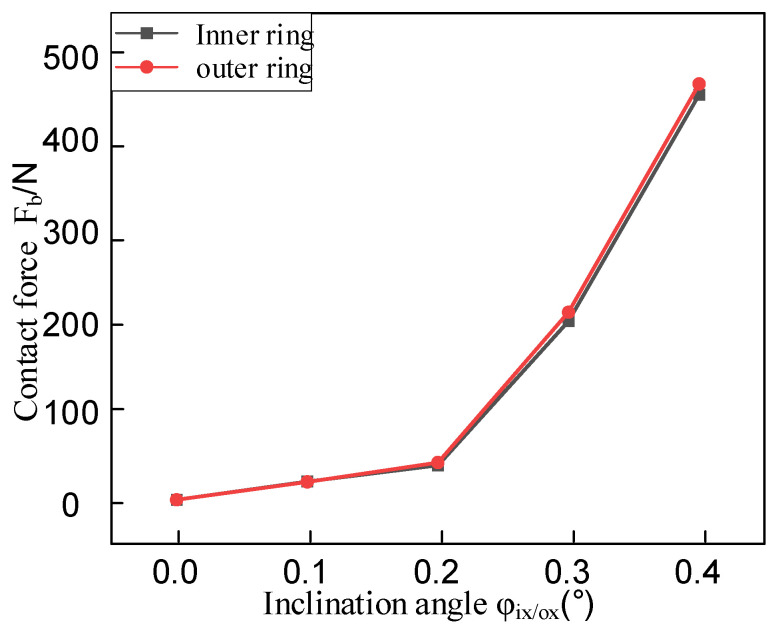
The bearing contact force varies with the amount of tilt misalignment.

Based on the maximum normal contact force calculation model derived from Hertzian contact theory, this study systematically investigates the influence of assembly misalignment on bearing contact mechanics. The numerical results in Figure 8 demonstrate that as the misalignment angle increases from 0.1° to 0.4°, both inner and outer ring misalignment induce a pronounced nonlinear growth characteristic in contact forces, with the growth gradient significantly intensifying with larger misalignment angles. Specifically, under the baseline condition without misalignment (φ_i/ox_ = 0°), the maximum normal contact force measures 3.745 N. When the inner ring misalignment angle φ_ix_ reaches 0.4°, the contact force surges to 473.919 N, representing a dramatic 126.5-fold increase compared to the baseline value. Such a magnitude of load fluctuation will substantially accelerate the bearing’s fatigue failure process. Comparative analysis reveals that the contact force amplitude under inner ring misalignment is approximately 8–12% lower than that caused by equivalent outer ring misalignment. This discrepancy primarily stems from the asymmetric geometric constraints between inner and outer rings.

### 3.3. Analysis of System Vibration Response Characteristics

**Figure 9:**
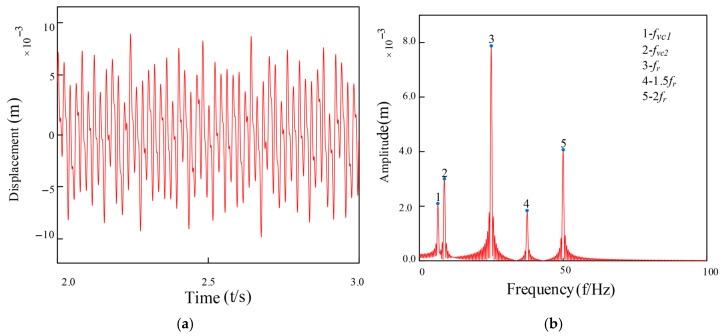
Health status: (**a**) vibration response signal; (**b**) FFT spectrum (post-FFT frequency spectrum).

To validate the accuracy and reliability of the established model, numerical simulations were conducted at identical operational parameters with a baseline rotational speed of 1500 rpm. Figure 9 presents the system’s dynamic response characteristics under healthy conditions, revealing complex nonlinear dynamic behaviors including periodic motion and period-doubling responses. Envelope demodulation analysis of the acceleration signals identifies the rotational frequency (f_r_ = 25 Hz) and its characteristic harmonic components (12.5 Hz, 37.5 Hz, 50 Hz). Spectral analysis results (Figure 9) demonstrate that the system’s primary frequency components comprise: f_r_ and its harmonics, bearing 1’s varying compliance vibration frequency (f_vc1_), and bearing 2’s varying compliance vibration frequency (f_vc2_). Amplitude comparison analysis confirms that the vibration energy at rotational frequency f_r_ dominates significantly, verifying the typical dynamic characteristic that rotor mass unbalance serves as the primary vibration source in fault-free operating conditions.

**Figure 10:**
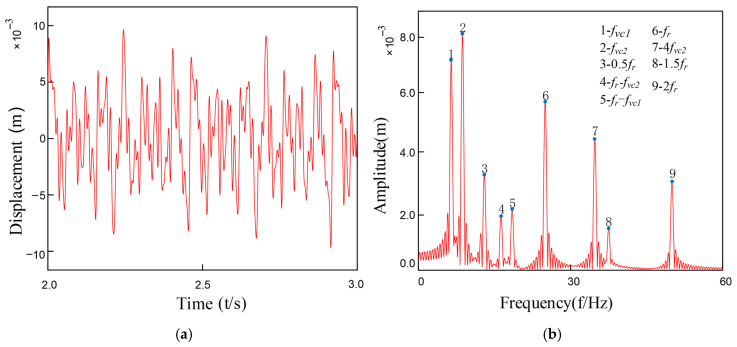
Bearing outer ring tilt misalignment: (**a**) Outer Ring Tilt Misalignment; (**b**) FFT Spectrum Plot.

**Figure 11:**
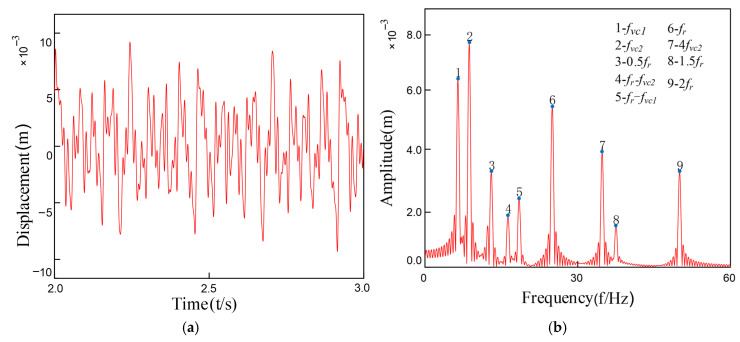
Bearing inner ring tilt misalignment: (**a**) Inner Ring Tilt Misalignment; (**b**)FFT Spectrum Plot.

Under coupled fault conditions, assembly misalignment was introduced at Bearing 1 (Node 5) with an outer ring misalignment angle φox = 0.2° and shaft tilt angle α = 0.1°. The nonlinear restoring force model was employed to simulate the faulty bearing characteristics, while Bearing 2 was normally installed at Node 7. Figure 10 and Figure 11 show the vibration response characteristics obtained by numerically solving the system dynamics equations. Spectral analysis results indicate that under this fault condition, the system exhibits the following characteristic evolutions: 1. The rotor fundamental frequency (f_r_) and its harmonic components are preserved; 2. Significant amplitude amplification of the varying compliance vibration frequency (f_vc1_) and its harmonics in faulty Bearing 1; 3. A notable amplification effect is also observed in the f_vc2_ components of normal Bearing 2; 4. The varying compliance frequency components may surpass the rotational frequency to become the dominant vibration source.

Comparative analysis reveals that while the spectral characteristic trends induced by inner and outer ring tilt misalignment are similar, under identical misalignment magnitudes (φ_ix_ = φ_ox_), the system vibration amplitude under outer ring tilt conditions is on average 15–20% higher than that caused by inner ring tilt. This phenomenon arises because: according to the analytical relationships in Equations (19)–(30), identical tilt angles at inner and outer rings generate significantly different rates of contact angle variation and clearance changes, consequently producing approximately 1.8-fold asymmetry in the magnitude of nonlinear bearing forces.

**Figure 12:**
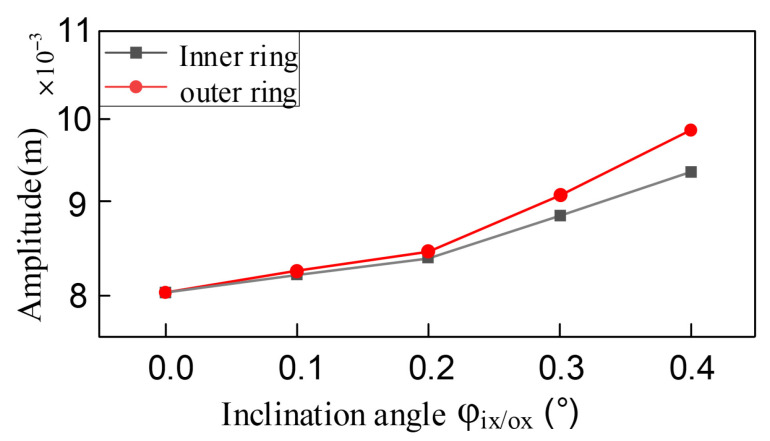
The amplitude of the system varies with the amount of tilt misalignment.

As the amplitude of the system vibration changes with the variation in the inclination misalignment amount of the inner and outer rings of the bearing, as shown in Figure 12, the system vibration amplitude shows a nonlinear upward trend with the inclination misalignment amount. Moreover, the change in the inclination angle of the outer ring has a greater impact on the system amplitude compared to that of the inner ring. As the inclination misalignment amount increases, the contact stiffness between the inner and outer rings of the bearing shows more intense periodic fluctuations, resulting in a decrease in the overall stiffness of the system and an exacerbation of its nonlinear characteristics. This leads to a significant increase in the response amplitude of the vibration system to external disturbances. At the same time, the impact of the inclination on the rolling elements and the raceways intensifies, the periodic excitation energy increases, and the proportion of energy absorbed and converted into vibration energy by the system rises, further causing the amplitude to increase.

**Figure 13:**
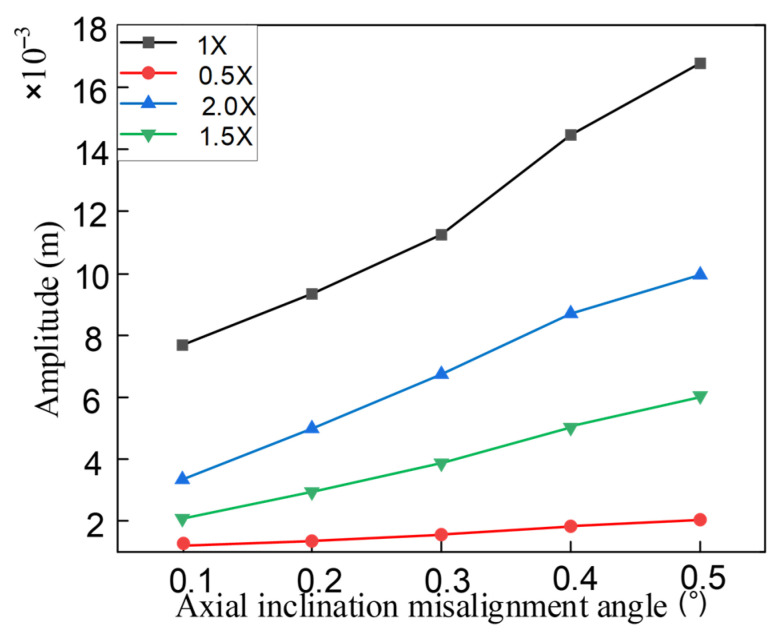
The amplitude of the harmonic frequency changes with misalignment.

The influence of the axial inclination misalignment angle on the system’s frequency characteristics was qualitatively analyzed. The quantitative impact of different degrees of misalignment on the harmonic frequencies of the system was also analyzed. Assuming that the inclination misalignment angle was changed from 0.1° to 0.5°, the trend of the system’s response amplitude with respect to the misalignment value was obtained, as shown in Figure 13. It can be seen that the amplitudes of all harmonic components increase with the increase in the misalignment angle, but the frequency increase of 1× and 2× is more obvious than that of 0.5× and 1.5×.

**Figure 14:**
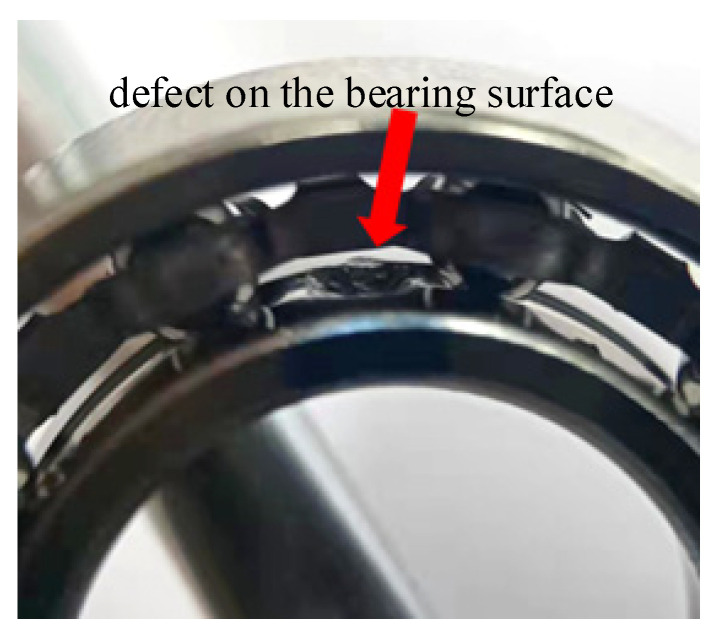
Realistic image of the defect on the bearing surface.

The defective bearing raceway surface includes waviness, roughness and irregularity in shape. However, the roughness and shape of the defective surface have a more significant impact on the local peeling defects on the bearing raceway. Figure 14 shows the real defect image of the bearing surface, with obvious irregularities along the edge of the defect. Existing studies usually simplify the defect into triangles, rectangles or squares, and derived the displacement excitation function of the raceway during the raceway movement. Moreover, the defect surface shows inhomogeneity, resulting in high-frequency vibration, which is a phenomenon often overlooked in previous studies.

### 3.4. Modeling of Rolling Bearing Outer Ring Faults and Response Analysis

**Figure 15:**
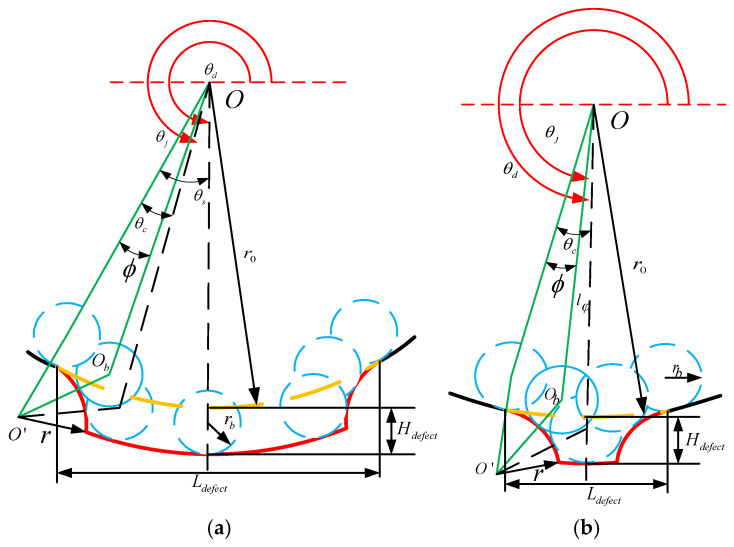
Supplementary displacements associated with outer ring faults: (**a**) Major fault contact; (**b**) Minor fault contact.

The precise characterization of the interaction between the rolling elements and the defect area significantly affects the vibration response of the dynamic model of the faulty bearing. To characterize the edge of the defect, this study adopts an arc segment with a radius of r, which conforms to the edge of the defect and allows for the adjustment of r to modify the properties of the edge. As shown in Figure 15, the red curve represents the shape of the defect, where L_defect_ and H_defect_ denote the geometric dimensions of the defect, the yellow curve represents the healthy trajectory, θ_d_ indicates the initial position of the defect center, O and O_b_, respectively, represent the centers of the outer ring and the roller, and O′ represents the center of the defect edge with a radius of r.

The supplementary displacement associated with the defect on the outer raceway can be described as follows:

(23) $$\delta =l_{\varphi }+r_{b}-r_{o}$$

As shown in Figure 15a, in the triangle ΔOO′O_b_, l_φ_ can be obtained using the cosine theorem:

(24) $${\bar{OO}}^{2}+{\bar{OO_{b}}}^{2}-{\bar{OO_{b}}}^{2}=2{\bar{OO}}^{\prime }\times \bar{OO_{b}}\times \cos\varphi$$

In the formula: $\bar{OO\prime }=r_{o}+r,\bar{OO_{b}}=l_{\varphi },\bar{O_{b}O}=r+r_{b}$.

Express it as a piecewise function:

(25) $$l_{\varphi }=\left\{\begin{array}{c} A+\varphi B=\theta_{j}+\theta_{s}-\theta_{d},\theta_{d}-\theta_{s}\leq \mathrm{mod}\left(\theta_{j},2\pi \right)\leq \theta_{d}+\theta_{c}-\theta_{s}\\ r_{o}+H_{dofcc}-r_{b}\;\theta_{d}+\theta_{c}-\theta_{s}<\mathrm{mod}\left(\theta_{j},2\pi \right)<\theta_{d}+\theta_{s}-\theta_{c}\\ A+\varphi B=-\theta_{j}+\theta_{s}+\theta_{d},\theta_{d}+\theta_{s}-\theta_{c}\leq \mathrm{mod}\left(\theta_{j},2\pi \right)\leq \theta_{d}+\theta_{s}\\ r_{o}-r_{b}\;otherwise \end{array}\right.$$

In the formula, A = $\sqrt{{\left(r+r_{b}\right)}^{2}-{\left(\left(r_{o}+r\right)\sin\varphi \right)}^{2}}$, B = $\left(r_{o}+r\right)\cos\varphi$, θ_s_ represents half of the angle formed by the defect relative to the center of the outer hub, while θ_s_ represents the angle between the rolling element that just contacts the edge of the defect and the rolling element that just contacts the bottom of the defect. These angles can be determined using the following equations, respectively:

(26) $$\theta_{s}=\arcsin\left(\frac{L_{defect}}{2r_{o}}\right)$$

(27) $$\theta_{c}=\arccos\left(\frac{{\left(r_{o}+r\right)}^{2}+{\left(r_{o}+H_{defect}-r_{b}\right)}^{2}-{\left(r_{b}+r\right)}^{2}}{2(r_{o}+r)(r_{o}+H_{defect}-r_{b})}\right)$$

Therefore, δ can be obtained as follows:

(28) $$\delta =\left\{\begin{array}{c} A+B+r_{b}-r_{o}\varphi =\theta_{j}+\theta_{s}-\theta_{d},\theta_{d}-\theta_{s}\leq \mathrm{mod}(\theta_{j},2\pi )\leq \theta_{d}+\theta_{c}-\theta_{s}\\ H_{defect}\;\theta_{d}+\theta_{c}-\theta_{s}\leq \mathrm{mod}(\theta_{j},2\pi )\leq \theta_{d}+\theta_{s}-\theta_{c}\\ A+B+r_{b}-r_{o}\varphi =-\theta_{j}+\theta_{s}+\theta_{d},\theta_{d}+\theta_{s}-\theta_{c}\leq \mathrm{mod}\left(\theta_{j},2\pi \right)\leq \theta_{d}+\theta_{s}\\ 0\;\mathrm{otherwise} \end{array}\right.$$

In Figure 15b, when the roller just touches the bottom of the defect, we know that θ_c_ = θ_s_. Then, we can determine the relationship between L_defect_ and H_defec_ as:

(29) $$\begin{array}{cc} L_{defect} & =2r_{o}\sin\theta_{s}=2r_{o}\sqrt{1-\cos^{2}\theta_{c}}\\ & =2r_{o}\sqrt{1-{\left(\frac{{\left(r_{o}+r\right)}^{2}+{\left(r_{o}+H_{defect}-r_{b}\right)}^{2}-{\left(r_{b}+r\right)}^{2}}{2(r_{o}+r)\left(r_{o}+H_{defect}-r_{b}\right)}\right)}^{2}} \end{array}$$

Therefore, when $L_{defect}<2r_{o}\sqrt{1-{\left(\frac{{\left(r_{o}+r\right)}^{2}+{\left(r_{o}+H_{defect}-r_{b}\right)}^{2}-{\left(r_{b}+r\right)}^{2}}{2(r_{o}+r)(r_{o}+H_{defect}-r_{b})}\right)}^{2}}$, if the ball does not come into contact with the bottom of the raceway, in this case, the time-varying displacement excitation function δ is:

(30) $$\delta =\left\{\begin{array}{c} A+B+r_{b}-r_{o}\varphi =\theta_{j}+\theta_{s}-\theta_{d},\theta_{d}-\theta_{s}\leq \mathrm{mod}\left(\theta_{j},2\pi \right)\leq \theta_{d}\\ A+B+r_{b}-r_{o}\varphi =-\theta_{j}+\theta_{s}+\theta_{d},\theta_{d}\leq \mathrm{mod}\left(\theta_{j},2\pi \right)\leq \theta_{d}+\theta_{s}\\ 0\;otherwise \end{array}\right.$$

**Figure 16:**
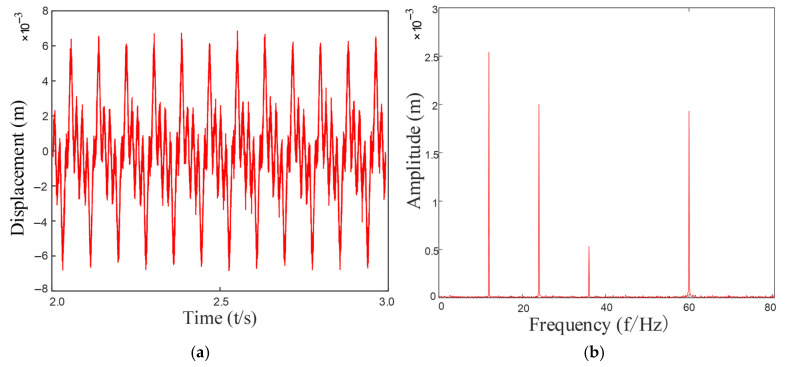
Vibration response of an outer ring fault: (**a**) Outer ring fault vibration response signal; (**b**) The frequency spectrum after FFT.

Based on the above formula, by substituting into the established dynamic equation of the rotary joint system, the vibration response of the system can be obtained as shown in Figure 16. After performing a Fourier transformation on it, the frequency components in the system can be observed in the figure. Various harmonic components and combined frequencies can be seen.

As can be seen from Figure 16a, the vibration signal exhibits obvious periodic impact characteristics, and the signal waveform presents a regular modulation pattern, indicating that a significant impact response was generated when the rolling elements passed through the faulty area of the outer ring. This impact reflects the periodic changes in system stiffness and contact conditions during the repeated contact process between the rolling elements and the stationary outer ring fault point.

Complexity analysis based on fuzzy entropy provides important supplementary information for these periodic characteristics. As a nonlinear dynamic parameter for measuring the complexity and irregularity of time series, the value of fuzzy entropy directly reflects the degree of randomness in the signal: a higher entropy value indicates a greater proportion of random components and weaker regularity in the signal, while a lower entropy value signifies stronger regularity and fewer random components.

From the frequency domain graph Figure 16b, significant characteristic frequency components can be observed, especially in the low-frequency range, presenting obvious main frequency peaks and their harmonics, mainly corresponding to the characteristic frequencies of the outer ring fault and their multiples. Under the background of a fixed structure of the outer ring, the relative contact frequency between the fault point and the rolling element is constant, so the characteristic frequency components in the spectral graph are stable and clear and are important frequency features for judging the defect of the outer ring. These frequency-domain characteristics are consistent with the results of the fuzzy entropy analysis. The fuzzy entropy value of the original fault signal is 0.6261, indicating that the signal contains both periodic impact components and substantial random noise interference. After noise reduction processing, the fuzzy entropy value of the filtered signal decreases significantly to 0.0173. This remarkable change demonstrates that the higher entropy value of the original signal primarily originates from noise-induced randomness, while the lower entropy value after noise reduction confirms that the fault impacts themselves exhibit high periodicity and regularity.

Furthermore, the spectrum rarely shows obvious modulation sideband components, which is related to the stationary state of the outer ring. Therefore, the modulation effect of the signal is weaker compared to the inner ring failure or the cage failure, and the spectral energy distribution is more concentrated, making it easier to extract features. The decrease in fuzzy entropy value further confirms the reliability of this periodicity from the perspective of information complexity. Research demonstrates that integrating fuzzy entropy with traditional time-frequency analysis methods can enhance the detection capability for incipient faults and weak signals, thereby providing more reliable technical means for equipment condition monitoring.

### 3.5. Modeling of Rolling Bearing Inner Ring Faults and Response Analysis

**Figure 17:**
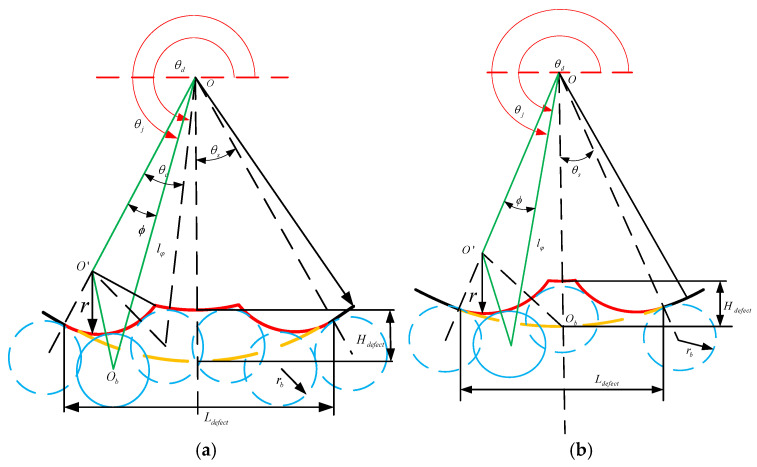
Supplementary displacement associated with an inner ring fault: (**a**) Major fault contact; (**b**) Minor fault contact.

During the operation of the bearing, the inner ring rotates synchronously with the main shaft. When there are defects on the surface of the inner ring’s raceway, the periodic impact loads generated by the rolling elements passing through the faulty area exhibit non-steady-state characteristics. Specifically, these are: 1. Random changes in contact conditions: The contact geometric conditions and force directions in each collision are different; 2. Fluctuations in load amplitude: The impact energy varies with the different contact positions; 3. Signal modulation effect: The amplitude of the vibration response is directly modulated by the load changes. This fault excitation characteristic is shown in Figure 17. Its physical mechanism can be attributed to the dynamic interaction between the geometric shape of the defect and the motion trajectory of the rolling elements. When the rolling elements pass through the defect area of the inner ring, due to the instantaneous change in contact constraints, a wide-frequency impact response with amplitude modulation will be generated. This feature can serve as an important basis for diagnosing the inner ring fault.

If there is a local failure on the inner raceway, the location of the defect on the inner raceway will rotate along with the shaft. The position can be calculated:

(31) $$\theta_{d}=\theta_{i}+\omega t$$

In this equation, θ_i_ represents the initial position of the inner defect.

Figure 17a shows a diagram depicting the movement of the roller across the fault. The supplementary displacement can be expressed as:

(32) $$\delta =-l_{\varphi }+r_{b}+r_{i}$$

In Figure 17b, when the roller just comes into contact with the bottom of the faulty inner ring, the geometric relationship can be used to derive that the relationship between L_defect_ and H_defect_ is:

(33) $$L_{defect}=2r_{i}\sin\theta_{s}=2r_{i}\sqrt{1-\cos^{2}\theta_{c}}$$

Therefore, only when $L_{defect}\geq 2r_{i}\sqrt{1-\cos^{2}\theta_{c}}$ similar to the outer ring, at this point, lφ can be expressed as:

(34) $$l_{\varphi }=\left\{\begin{array}{c} C+\varphi D=\theta_{j}+\theta_{s}-\theta_{d},\theta_{d}-\theta_{s}\leq \mathrm{mod}(\theta_{j},2\pi )\leq \theta_{d}+\theta_{c}-\theta_{s}\\ r_{i}-H_{defect}+r_{b}\;\theta_{d}+\theta_{c}-\theta_{s}<\mathrm{mod}(\theta_{j},2\pi )<\theta_{d}+\theta_{s}-\theta_{c}\\ C+\varphi D=-\theta_{j}+\theta_{s}+\theta_{d},\theta_{d}+\theta_{s}-\theta_{c}\leq \mathrm{mod}(\theta_{j},2\pi )\leq \theta_{d}+\theta_{s}\\ r_{i}+r_{b}\;otherwise \end{array}\right.$$

In the formula C = $\sqrt{{\left(r+r_{b}\right)}^{2}-{\left(\left(r_{i}-r\right)\sin\varphi \right)}^{2}}$, D = $\left(r_{i}-r\right)\cos\varphi$, the expressions of θc and θs can be given as follows:

(35) $$\theta_{c}=\arccos\left(\frac{{\left(r_{i}-r\right)}^{2}+{\left(r_{i}-H_{defect}+r_{b}\right)}^{2}-{\left(r_{b}+r\right)}^{2}}{2(r_{i}-r)(r_{i}-H_{defect}+r_{b})}\right)$$

(36) $$\theta_{s}=\arcsin\left(\frac{L_{defect}}{2r_{i}}\right)$$

δ can be expressed as:

(37) $$\delta =\left\{\begin{array}{c} r_{i}+r_{b}-C-\varphi D=\theta_{j}+\theta_{s}-\theta_{d},\theta_{d}-\theta_{s}\leq \mathrm{mod}\left(\theta_{j},2\pi \right)\leq \theta_{d}+\theta_{c}-\theta_{s}\\ H_{defect}\;\theta_{d}+\theta_{c}-\theta_{s}<\mathrm{mod}(\theta_{j},2\pi )<\theta_{d}+\theta_{s}-\theta_{c}\\ r_{i}+r_{b}-C-\varphi D=-\theta_{j}+\theta_{s}+\theta_{d},\theta_{d}+\theta_{s}-\theta_{c}\leq \mathrm{mod}(\theta_{j},2\pi )\leq \theta_{d}+\theta_{s}\\ 0\;otherwise \end{array}\right.$$

when $L_{defect}<2r_{i}\sqrt{1-\cos^{2}\theta_{j}}$, δ can be expressed as:

(38) $$\delta =\left\{\begin{array}{c} r_{i}+r_{b}-C-\varphi D=\theta_{j}+\theta_{s}-\theta_{d},\theta_{d}-\theta_{s}\leq \mathrm{mod}(\theta_{j},2\pi )\leq \theta_{d}\\ r_{i}+r_{b}-C-\varphi D=-\theta_{j}+\theta_{s}+\theta_{d},\theta_{d}\leq \mathrm{mod}(\theta_{j},2\pi )\leq \theta_{d}+\theta_{s}\\ 0\;\mathrm{otherwise} \end{array}\right.$$

Based on the above analysis, the time-varying displacement excitation model of the rolling elements when passing through the defects of the outer and inner rings can be expressed as follows:

(39) $$\delta_{j}=(x_{o}-x_{i})\cos\theta_{j}+(y_{0}-y_{i})\sin\theta_{j}-C-\delta$$

**Figure 18:**
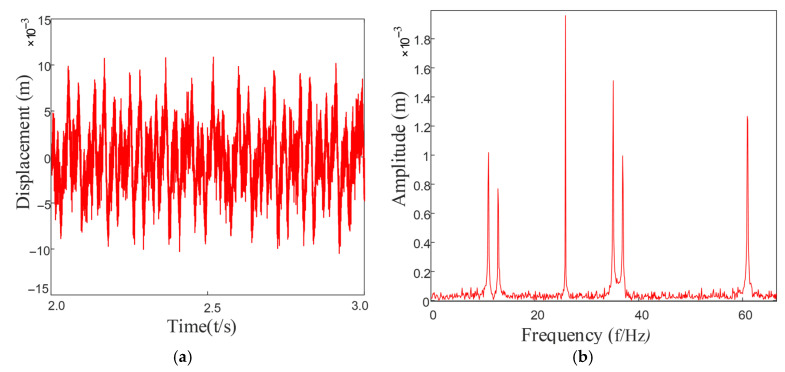
Schematic diagram of the inner ring fault: (**a**) Inner ring fault vibration response signal; (**b**) The frequency spectrum after FFT.

Based on the above formula, by substituting into the established dynamic equation of the rotary joint system, the vibration response of the system can be obtained as shown in Figure 18. After performing a Fourier transformation on it, the frequency components in the system can be observed in the figure. Various harmonic components and combined frequencies can be seen.

As can be seen from Figure 18, the signal as a whole exhibits strong non-stationarity and modulation characteristics. The vibration amplitude shows a trend of increasing periodicity over time, indicating that periodic impact responses were generated when the rolling elements passed through the faulty area of the inner ring.

In Figure 18b, multiple main frequency peaks and their harmonic components can be clearly observed, with the spectral peaks in the medium and low frequency bands being particularly prominent. The spectral characteristics of the inner ring fault mainly manifest as obvious spectral peaks at the characteristic frequency fi and its harmonics nf_i_. Additionally, it can be observed that the transverse frequency f_r_ and the modulation frequency f_r_ ± nf_i_ related to the inner ring’s frequency components are also present, further confirming that the fault generates periodic impacts and that the fault influence forms a modulation phenomenon in the frequency domain over time.

The complexity analysis based on fuzzy entropy further reveals the dynamic characteristics of the inner race fault signal. The fuzzy entropy value of the original noise-contaminated fault signal is 1.3887, significantly higher than the value of 0.0213 for the noise-free reference signal. This notable difference indicates that although the original fault signal exhibits high complexity and randomness due to noise interference, the intrinsic fault impact components actually possess a high degree of periodicity. The substantial decrease in entropy value clearly demonstrates the dominant influence of noise on signal complexity, while the extremely low entropy value after noise reduction confirms that the inner race fault impacts themselves exhibit strong regularity and predictability.

The spectrum of the inner ring fault signal exhibits greater energy concentration and a richer frequency composition, indicating that the fault has altered the inherent dynamic characteristics of the system. In particular, it plays a decisive role in the response of the rolling elements when passing through the defect area of the inner ring. Meanwhile, the fuzzy entropy metric further reveals variations in the complexity of the fault signal under noise interference. Fuzzy entropy analysis provides an effective supplementary basis for the diagnosis of inner race faults from the perspective of information complexity, offering a distinct advantage over traditional time-frequency analysis methods.

## 4. Random Roughness Characterization Method Based on Gaussian Filtering

Authors In this section, in order to address the unevenness observed on the surface of local defects, we utilize the Gaussian filter to generate the local defect roughness, simulating the actual surface morphology of the defect. The detailed program overview is as follows:

(1) Generate a random matrix with a mean of 0, which can be expressed as:

(40) $$\begin{array}{cc} R & =\sigma \times randn(N,N)\;\\ & =\sigma \times \left[\begin{array}{cccc} a_{11} & a_{12} & \ldots & a_{1N}\\ a_{21} & a_{22} & \ldots & a_{2N}\\ \vdots & \vdots & \vdots & \vdots \\ a_{N1} & a_{N2} & \ldots & a_{NN} \end{array}\right] \end{array}$$

Here, σ is used to control the magnitude of the generated roughness, and N refers to the dimension of the random matrix.

(2) Define a Gaussian filter function, which represents the feature with a specific width and height of roughness, to create the Gaussian filter matrix G. The general form of the two-dimensional Gaussian distribution function can be expressed as:

(41) $$G(x,y)=\frac{1}{2\pi \sigma_{x}\sigma_{y}}e^{-\frac{{\left(x-\mu_{x}\right)}^{2}+{\left(y-\mu_{y}\right)}^{2}}{2\sigma_{x}\sigma_{y}}}$$

In the equation, when μ_x_ = μ_y_ = 0 and σ_x_ = σ_y_ = σ, the Gaussian distribution function is:

(42) $$G(x,y)=\frac{1}{2\pi \sigma^{2}}e^{-\frac{x^{2}+y^{2}}{2\sigma^{2}}}$$

(3) Perform convolution between the random matrix R and the Gaussian filtering matrix G to obtain the surface morphology matrix of the bearing raceway defect:

(43) $$D^{\prime }=R\times G$$

(4) By slicing, the required local defect roughness curve is obtained, and it is superimposed with the defect excitation function to create the local defect morphology curve.

(44) $$\delta_{j}=(x_{o}-x_{i})\cos\theta_{j}+(y_{0}-y_{i})\sin\theta_{j}-C-\delta -x$$

When the roller comes into contact with the rough surface of the defect, it enters the micro-contact domain. The Hertz contact theory cannot be directly applied in this case. The micro-contact model conceptualizes the interaction of the rough surfaces as similar to the contact between a smooth plane and the corresponding equivalent rough surface. They proposed a simplified micro-rough contact model to study the microscopic contact mechanism of the mating surfaces. The stiffness of the rough contact interface can be determined using the formula given in the literature.

(45) $$k_{t}^{\prime }=2NE\beta^{1/2}\sqrt{\frac{\sigma }{2\pi }}\int_{h_{n}-d_{n}}^{\infty }{\left(z_{n}+(h_{n}-d_{n})\right)}^{1/2}e^{-\frac{z_{n}^{2}}{2}}dz_{n}$$

**Figure 19:**
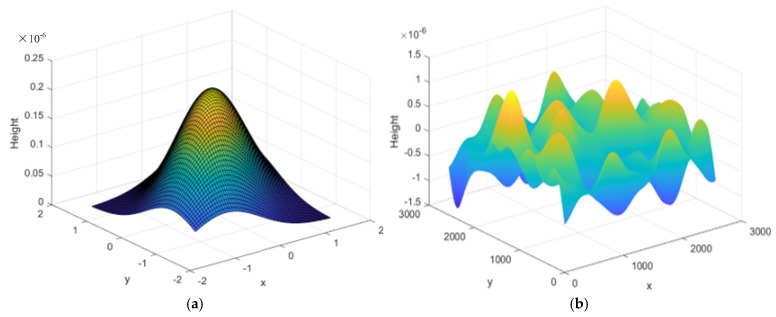
Simulation results of defect surface morphology based on Gaussian filter: (**a**) Gaussian filter matrix (The figure is a filtering tool used for generating contours. The color represents the weight level. The change in color (from blue at the edge to yellow at the center) indicates the increasing influence of the color on the surface topography); (**b**) Surface contour matrix (The figure represents the final generated defect surface itself. The color directly indicates the actual peaks and valleys of the surface. The change in color (from blue to green to yellow) represents the gradual increase in the physical height of the surface from the bottom of the valley to the peak).

The variables in the formula are defined as follows: N represents the total number of micro-convex bodies on the rough surface, E represents the equivalent elastic modulus of the contact interface, β represents the curvature radius of each rough unit, z_n_ represents the height of the rough micro-convex body, and h_n_ − d_n_ represents the measured value of the average distance of the separation between the rough surfaces. The height of the rough micro-convex body follows a Gaussian distribution with a specific standard deviation. The simulation results are shown in Figure 19, which verifies the effectiveness of this method in generating local defect roughness curves. By superimposing the generated roughness curve with the defect excitation function, we can create a local defect morphology curve that is closer to the actual working condition, thereby providing a more reliable foundation for the dynamic characteristic analysis of bearing defects.

## 5. Model of Composite Fault Vibration Signals for Internal Bearings of Rotary Joint

The composite failure of rolling bearings is essentially a phenomenon where multiple failure mechanisms work together. Systematically studying the dynamic impact response characteristics under such coupled failure conditions is of great value for improving the failure mechanism theory and guiding engineering practice. The rotating joint studied in this paper adopts a unique “single rotor—double support bearing” structural design. The rotating components and the stationary housing are assembled and connected through interference fit. This rigid connection method is prone to inducing axial misalignment between the two support bearings, thereby forming a typical “bearing defect—misalignment” composite failure dynamic system. Based on the different failure occurrence positions, this study mainly considers the following three typical composite failure modes: 1. Coupling model of bearing outer ring failure and misalignment; 2. Coupling model of bearing inner ring failure and misalignment; 3. Coupling model of bearing inner and outer rings composite failure and misalignment. The analysis of these composite failure modes will provide an important theoretical basis for the subsequent fault diagnosis of rotating joints.

Based on the established system dynamics theoretical framework mentioned above, this study solved the model by developing a MATLAB 2024 numerical calculation program. The specific implementation process included: Parameter input: 1. The variation in contact angles under different coupled fault conditions; 2. The dynamic change values of bearing working clearance; 3. Nonlinear contact forces and other key parameters; Embedding dynamic differential equations; 4. Numerical solution: Use a variable-step ODE solver to obtain the time-domain vibration responses of the system under various fault degrees; 5. Signal processing: Perform spectral analysis on the vibration signals obtained from the simulation and extract characteristic frequency components; Taking the double-bearing-single-rotor system established as the research object, set the fixed rotational speed at 1500 rpm, the skew angle φi/ox = 0.2°, and the axis deflection angle α = 0.1°. To improve data processing efficiency and ensure analysis reliability, the vibration signals after the system enters the steady state for 2–3 s were specially selected as the research samples.

**Figure 20:**
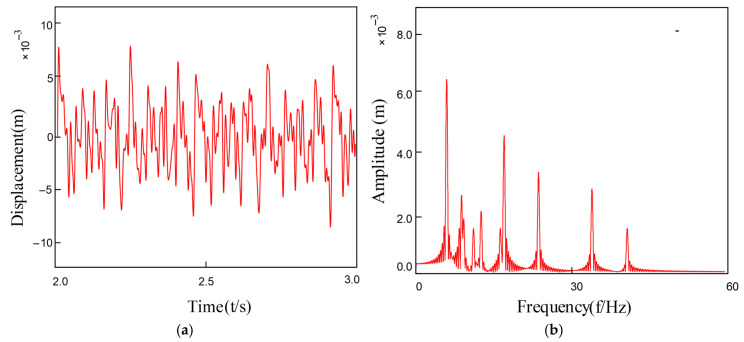
The outer ring is faulty and tilted: (**a**) Vibration signal of outer ring defect with tilt misalignment; (**b**) FFT Spectrum.

**Figure 21:**
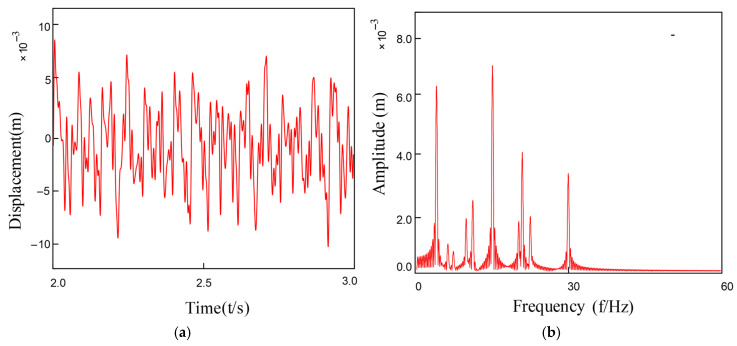
The inner ring is faulty and tilted out of alignment: (**a**) Inner ring defect with tilt misalignment vibration signa; (**b**) FFT Spectrum.

**Figure 22:**
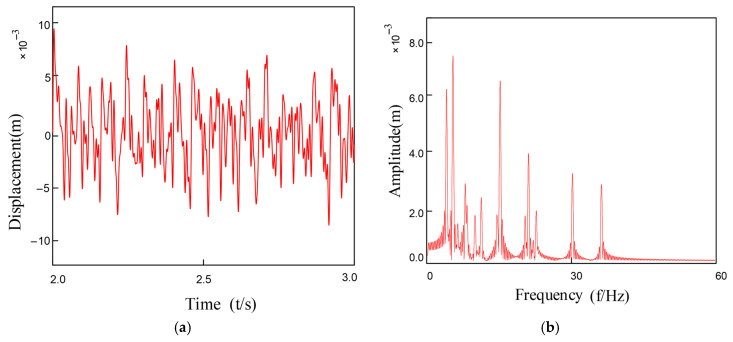
The inner and outer rings are faulty and tilted out of alignment: (**a**) Inner and outer ring defects with tilt misalignment vibration signal; (**b**) FFT Spectrum.

As can be seen from Figure 20, Figure 21 and Figure 22, through spectral analysis, the following characteristic frequency components and their modulation products can be clearly identified: 1. Outer ring fault characteristics: the fundamental frequency fo and its harmonics nfo (n = 1, 2, 3…); 2. Fault characteristics of the inner ring: fundamental frequency fi and its sidebands nfi; 3. Frequency related to misalignment: vibration frequency fvc1 of the flexible bearing 1 and its harmonics, modulation component of the flexible bearing 2 vibration frequency fvc2, combined modulation components among these frequencies; 4. Dynamic characteristics of the rotor: rotational frequency fr and its higher-order harmonics. Under the combined effect of multiple interference sources, the bearing fault signals often exhibit typical weak characteristics, and their impact response components are easily obscured by strong background noise. Specifically, the amplitude of the fault characteristic frequency is significantly lower than the dominant harmonic components. In the time-domain signal, it presents a low signal-to-noise ratio characteristic, and in the frequency domain, the characteristic frequency is masked by the modulated harmonics. This formation of weak fault characteristics mainly results from the combined action of multiple-source vibration coupling, wide-band distribution of fault impact energy, and enhancement within the background noise band. The signals of the inner ring and the rolling elements are affected by the interference sources, and the frequency amplitudes of the inner ring and the rolling elements are very low. Through the envelope spectrum, it can be seen that the modulation frequency of the gear is much higher than the fault frequency of the bearing, and the fault characteristics in the high-frequency region are submerged in the interference signal and cannot be accurately identified.

When observing the peak characteristics of the time-domain signal, it can be found that: when there is only a single failure in the inner ring or the outer ring, the amplitude of the impact response is smaller, and the peak is relatively lower. However, when the failure form becomes more complex and multiple types of coupled failures occur, the amplitude of the signal significantly increases. Especially in the case of a composite failure, its peak is much higher than in the case of a single failure, indicating that the impact energy is stronger.

## 6. Test Verification

To verify the validity of the multi-fault coupling dynamic model of the internal double bearings-single rotor system of the rotary joint, which was established in the previous text, as well as the vibration response simulation method, this study relied on the online monitoring platform designed and developed based on multi-sensor fusion. A series of experimental validations were designed and implemented. By constructing test schemes with different fault levels and multiple fault types, the vibration response signals during the operation of the rotary joint were systematically collected. The aim was to compare the simulation results of the model with the actual vibration characteristics, thereby verifying the accuracy and applicability of the proposed theoretical model.

### 6.1. Experimental Protocol Design

In order to accurately simulate the fault characteristics of bearings under actual working conditions, this study introduces typical damages into the interior of the bearings through artificial fault settings. The basic parameters of the selected bearings are shown in Table 2. Fault defects with clear geometric dimensions were preset on the inner and outer ring surfaces of the bearings [32]. The fault size parameters are detailed in Table 3. In addition, considering the nonlinear response characteristics of the system, during the test process, the working condition parameters (such as load, speed, etc.) were ensured to be stable and controllable to minimize the influence of environmental noise and external disturbances on the test data, thereby improving the usability of the test data and the rigor of the comparative analysis.

For better subsequent analysis, in the experiment, the inclination angle of the inner and outer rings φ_i/ox_ = 0.2°, and the axial inclination misalignment angle α = 0.1°. A fault occurred in the bearing inner ring near the motor end, while the other was a healthy bearing. Separate tests were conducted for the bearing outer ring fault and axial misalignment, the bearing inner ring fault and axial misalignment, and the combined fault of the bearing inner and outer rings and axial misalignment.

### 6.2. Data Collection and Analysis of Test Results

According to the test plan and test item parameters designed in Section 6.1, using an acceleration sensor, a 10 s time signal is collected every 5 min. The motor operates at 1500 rpm, and the sampling rate is set at 25.6 kHz to ensure the complete capture of high-frequency components. The vibration signals collected during the test are subjected to Fourier transformation and analyzed. The time domain graph and envelope spectrum graph are shown in the following figure.

**Figure 23:**
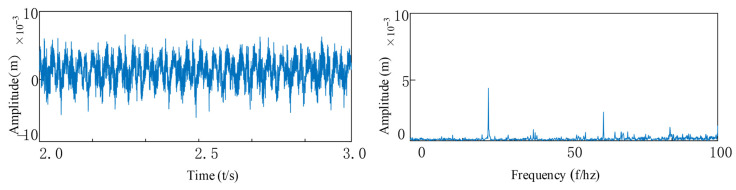
Bearing health.

Under fault-free conditions, as shown in Figure 23, the vibration signal of the rotary joint is dominated by high-frequency noise. The inherent frequency characteristics of the bearing are difficult to identify in the envelope spectrum, and the amplitude is relatively weak.

**Figure 24:**
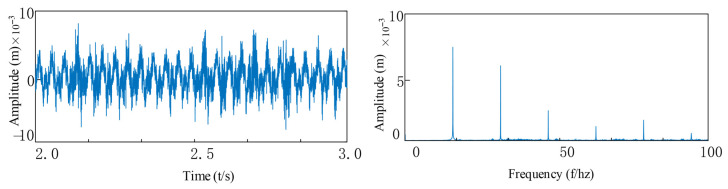
Medium outer ring failure and tilt misalignment.

**Figure 25:**
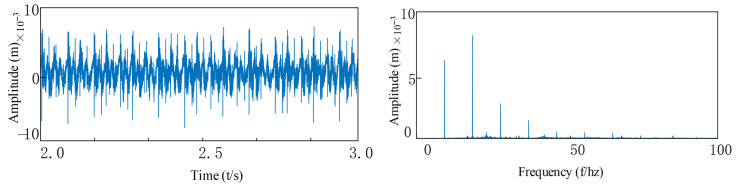
Medium inner ring failure and tilt misalignment.

**Figure 26:**
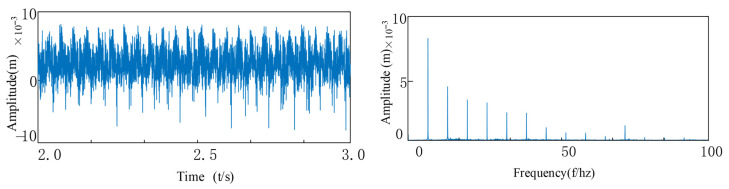
Medium inner and outer ring faults and tilt misalignment.

Referring to Figure 24, Figure 25 and Figure 26, when multiple consecutive failures occur in the double bearings inside the rotary joint:

From the envelope spectrogram, it can be seen that multiple characteristic frequencies appear in the envelope graph, and the amplitude is approximately doubled compared to that of a healthy bearing. Under the composite fault condition, the spectrum presents a dense distribution of characteristic frequencies, and the amplitude is significantly higher than that of the single fault mode. The envelope spectrogram of the experimental data can clearly identify the characteristic frequencies of the rotational frequency, the inner and outer ring faults of the bearing, and their harmonics. At the same time, the vibration characteristics of VC (Varying Compliance) caused by misalignment are observed. Under the condition of consistent usage object and operating conditions, It is worth noting that the simulation results are generally higher in amplitude than the experimental data, which is mainly due to the influence of the bearing damping effect in the actual system, resulting in inherent differences between the simulation and the measurement.

**Figure 27:**
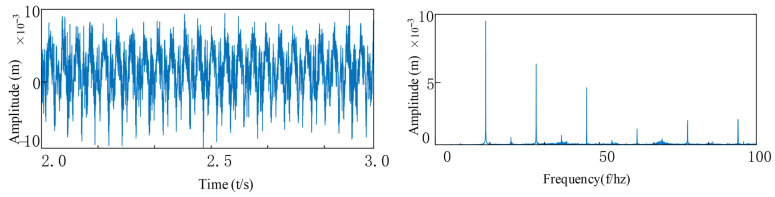
Severe outer ring failure and tilt misalignment.

**Figure 28:**
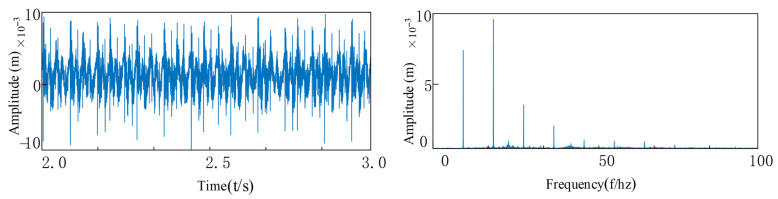
Severe inner ring failure and tilt misalignment.

**Figure 29:**
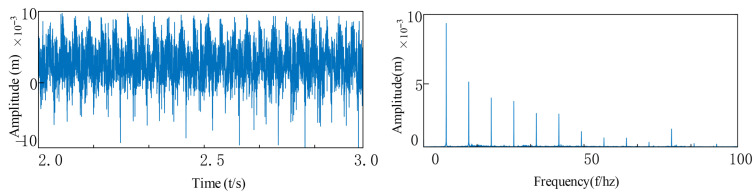
Serious inner and outer ring failure and tilt misalignment.

Under the same working conditions, the degree of bearing failure of the inner and outer rings was adjusted to be severe. As can be seen from Figure 27, Figure 28 and Figure 29, when the internal bearing of the rotary joint suffered a severe failure, the amplitude was approximately 10% higher compared to the moderate failure. The amplitude was consistent with the theoretical trend.

**Figure 30:**
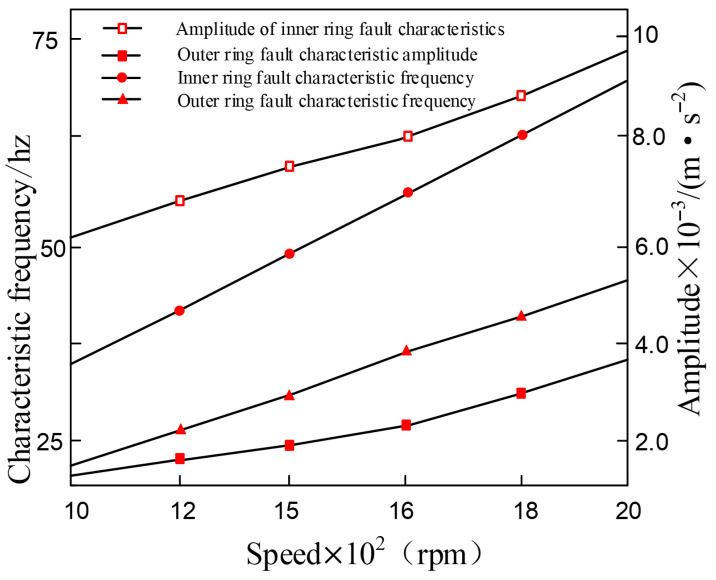
Vibration response frequency and amplitude of composite faults in the inner and outer rings at different speeds.

The operating stability of the bearing is significantly correlated with the rotational speed. This prompts us to investigate the dynamic response characteristics of the composite faulty bearing under variable rotational speed conditions. In this study, the size of the inner and outer ring faults was set at 0.3 mm, and the rotational speed was gradually increased from 1000 rpm to 2000 rpm. The rotational speed-dependent characteristics of the force on the rolling elements were not considered for the time being. To clearly represent the evolution law of vibration characteristics, Figure 30 shows the evolution trend of the characteristic frequencies and corresponding amplitudes of the composite fault during the variable rotational speed process.

The analysis results in Figure 30 indicate that the rotational speed of the bearing is positively correlated with the fault characteristic frequency of the inner and outer rings. As the rotational speed increases, not only does the fault characteristic frequency increase, but the corresponding vibration amplitude also significantly enhances. This confirms that an increase in rotational speed will intensify the dynamic excitation effect within the composite fault bearing. It is noteworthy that, due to the fixed position of the outer ring and the rotation of the inner ring with the shaft, the vibration response amplitude of the outer ring fault is always lower than that of the inner ring fault. This phenomenon indicates that as the rotational speed increases, the impact of the outer ring defect on the system vibration is relatively smaller. The study also found that the higher the rotational speed, the more significant the deterioration effect of the internal and external ring composite defects on the operating condition of the bearing.

## 7. Conclusions

This paper studies the dynamic characteristics of a double-support bearing–rotor system under tilt-misalignment coupling and composite faults. Through the combination of theoretical modeling, numerical simulation and experimental verification, a complete dynamic analysis system for the bearing–rotor system is established.

In the study of the inclined-non-aligned coupling condition, based on the Hertz contact theory, a nonlinear mechanical model of rolling body contact considering the inclination of the inner and outer rings of the bearing and the misalignment of the axis was established, revealing the variation laws of contact force distribution and the system’s force state. By introducing the axial misalignment additional load, the dynamic description of the double-support bearing–rotor system was improved, and the motion differential equation set containing the nonlinear time-varying contact stiffness characteristics was derived. The simulation results show that changes in the inclination angle and misalignment amount can significantly affect the system’s dynamic characteristics, manifesting as multiple harmonic components such as 0.5×, 1×, 1.5×, and 2× in the frequency domain, and the characteristic frequency amplitude increases with the increase in misalignment degree. It is particularly notable that when the misalignment is severe, the amplitude of the bearing VC frequency may exceed the rotational frequency, becoming the dominant excitation frequency.

In the study of the bearing fault mechanism, nonlinear dynamic models of single-point outer ring fault, inner ring fault, and composite fault states were constructed, and Gaussian filtering technology was introduced to simulate the surface roughness of the rolling body and the raceway, improving the model accuracy. Through numerical simulation, the influence of various faults on the spectral characteristics and the modulation law of characteristic frequencies was revealed, and it was found that harmonic interference would lead to a decrease in the amplitude of the characteristic frequency, an increase in non-stationarity, and characteristic aliasing phenomena, significantly increasing the complexity of fault diagnosis.

In the experimental verification aspect, a rotary joint fault simulation test platform was built, and through vibration data acquisition under different working conditions, the correctness of the theoretical model was verified. The experimental results not only confirmed the special vibration characteristics under the inclined-non-aligned coupling condition but also revealed the existence characteristics of characteristic frequency coupling and new modulation components in the composite fault state.

This study represents a theoretical advance, extending the analysis from single faults to composite faults and from ideal working conditions to complex coupling conditions The established dynamic model and simulation analysis methods provide an important foundation for subsequent research on the characteristics of composite faults and the development of intelligent diagnostic algorithms. The research results not only deepen the understanding of the complex dynamic behavior of the bearing–rotor system but also provide theoretical support and experimental basis for feature extraction and fault diagnosis of the rotary joint health monitoring system. The dynamic model established in this study primarily focuses on pure mechanical misalignment conditions and does not incorporate practical factors such as thermal effects, variations in lubrication conditions, bearing dynamic clearance, or nonlinear damping of couplings, which may significantly alter the system’s vibration response characteristics. Future research could consider conducting more extensive experimental tests that account for these factors to validate the model’s applicability to multiple fault types.

## Figures and Tables

**Table 1:** Rotor parameters and operating conditions.

Rotor	Numerical Value
Outer diameter d (mm)	8.5
Axial segment length L (mm)	230
Number of units N	10
Speed ω (rpm)	1000

**Table 2:** Bearing parameter table.

Bearings	Numerical Value
Outer track radius R_o_ (mm)	19.98
Inner track radius R_i_ (mm)	14.55
Number of rolling elements Z	8
Radial clearance c_0_/δ_r_ (μmm)	2
Roller length l (mm)	10

**Table 3:** Internal bearing failure parameters.

Fault Size	Medium	Serious
Inner ring	0.15 mm	0.3 mm
Outer ring	0.15 mm	0.3 mm

## Data Availability

These data are collected by the experiment. The code is written according to the proposed model.
